# Evaluation of Refraction Outcomes From the QuickSee Wavefront Autorefractor Versus Subjective Clinical Refractometry in Children With Restricted Access to Eye Care in Amazonas, Brazil

**DOI:** 10.7759/cureus.69945

**Published:** 2024-09-22

**Authors:** Claudio do Carmo Chaves Filho, Daniel Oliveira Dantas, Francisco Carlos de Castro Neto, Dillan Cunha Amaral, Ricardo Noguera Louzada, Milton Ruiz Alves

**Affiliations:** 1 Department of Clinical Surgery, Faculty of Medicine, Federal University of Amazonas, Manaus, BRA; 2 Division of Ophthalmology, Faculty of Medicine, University of São Paulo, São Paulo, BRA; 3 Department of Computer Science, Federal University of Sergipe, São Cristóvão, BRA; 4 School of Medicine, Estácio Medical School of Juazeiro, Juazeiro, BRA; 5 Ophthalmology, Faculty of Medicine, Federal University of Rio de Janeiro, Rio de Janeiro, BRA

**Keywords:** child, comparative study, elementary school, myopia, ocular, preschool, refraction

## Abstract

Background

This study compared the refraction measurements of the QuickSee wavefront autorefractor with those from clinical refraction measurements in preschool and elementary school children from public schools in Amazonas, Brazil.

Methodology

Refractometry was performed on 368 eyes from 368 healthy Brazilian public school students aged 4 to 7 years using both the QuickSee and subjective clinical methods under cycloplegia. Only right-eye data were analyzed. The results were converted into spherical equivalents and vector magnitudes for comparison.

Results

The difference in spherical equivalents between QuickSee and subjective clinical refractometry under cycloplegia was +0.38 ± 0.60 Diopters (D) (p < 0.0001). Along the 90° axis, the vector difference was +0.20 ± 0.33 D (p < 0.0001), and the oblique vector difference was +0.03 ± 0.22 D (p = 0.0395).

Conclusions

While a statistically significant difference was found between the QuickSee and subjective clinical refractometry results, the difference was not clinically meaningful. The strong agreement between these methods supports the utility of QuickSee as an effective tool for refractive assessment in children with limited access to eye care. These findings provide confidence in using QuickSee in public school settings as a reliable alternative for vision screening.

## Introduction

The primary reason for low vision in public preschool and elementary school students is uncorrected refractive error (URE) [1-5]. About 10% of the students have refractive errors that must be corrected, and 5% have visual acuity (VA) less than 50% of normal vision [6-8]. About 95% of ophthalmologic problems in schoolchildren are avoided or mitigated with eye health promotion and care [6]. Visual improvement, as well as methods aimed at promoting low vision and blindness, are among the most cost-effective health interventions for improving visual quality [9-12]. This study was conducted in Manaus, Amazonas, Brazil, where access to ophthalmological care is limited, particularly for children in public schools.

Currently, public preschool and elementary school students are not guaranteed access to the diagnosis and correction of URE, which causes visual impairment and impacts knowledge acquisition, school dropout and repetition, poor motor skills, difficulty in social interaction, and low self-esteem [8].

The World Health Organization (WHO) has highlighted the barriers represented by the acquisition of eyeglasses and encourages that priority be given to projects that identify and correct URE with the distribution of free or low-cost eyeglasses [6]. Given this scenario, there is a need to improve the management of human and financial resources involved in the visual and refractive screening of public preschool and elementary school students to increase its coverage and bring ophthalmological care to needy areas and those farther away from the country’s large urban centers. One of the ways to expand the ophthalmological care of public schoolchildren includes the incorporation of new technologies into this process, such as refractive screening with a low-cost portable QuickSee wavefront autorefractor (Plenoptika, USA) [13-16].

This study evaluated the accuracy of QuickSee refraction compared to subjective clinical refraction in preschool and elementary school children with limited access to eye care. It also assessed the reliability of QuickSee as a practical tool for detecting and correcting refractive errors in underserved areas.

## Materials and methods

The Ethics Committee of Getúlio Vargas University Hospital at the University of Amazonas in Manaus approved our research protocol (approval number: 75560623.0.0000.9167). A total of 638 school-age children were subjected to refractometry at their schools in Manaus, Amazonas, Brazil. Informed consent was obtained from the parents or legal guardians of all participants.

The participants were aged 4-7 years and included preschool and elementary students from public schools in Manaus, Amazonas, Brazil. Data on age, birth date, gender, and ophthalmological findings were collected. At the time of the ophthalmological evaluation, the schoolchildren did not present an active allergic, inflammatory, or infectious condition on the anterior ocular segment.

We conducted the ophthalmological examination in the following sequence: VA assessment at 5 m using a Snellen chart with no correction, followed by three static refraction measurements using the QuickSee device (Table 1) under cycloplegia, clinical refraction assessment under cycloplegia, including manual retinoscopy and use of refractor, VA testing with correction, and, finally, slit lamp examination and fundus evaluation. Cycloplegia was induced by instilling one drop of 1% cyclopentolate (Ciclopentolato 10 mg/mL, Allergan), followed by a drop of 1% tropicamide after five minutes (Mydriacyl 1%, Novartis) [17,18]. Refraction was performed 30 minutes after the initial instillation [19]. Essilor (Rio de Janeiro, Brazil) provided the QuickSee device (Table 1) used for this study, facilitating the research.

**Table 1:** Main features of the QuickSee handheld autorefractor.

Characteristics of the QuickSee autorefractor	
Dimensions	28 × 16.5 × 8.25 cm, 1 kg
Accommodation control	Light/target observation placed at 3 m
Measurement of spherical errors	±10.00 D, increments of 0.01 D, 0.125 D, and 0.25 D
Measurement of cylindrical errors	±6.00 D, increments of 0.01 D, 0.125 D, and 0.25 D
Measurement of axes	0–180°, increments of 1°, 5°, and 10°
Interpupillary distance	47–78 mm continuous
FDA status	Class I
Requirement of mydriasis/cycloplegia	No
Requirement of ambient lighting	No

Statistical analysis

Data collection focused solely on the right eye (RE) to eliminate any issues arising from the interdependence of measurements from both eyes of the same person. Refraction measurements from both techniques were averaged and compared. Spherical values were in diopters, cylindrical values in cylindrical diopters, and axes in degrees for refractive error assessment.

For statistical analysis and averaging, the measurements were converted to spherical equivalent (SE), determined by adding half of the astigmatism value to the spherical value.

Additionally, components of cylindrical were calculated as power vectors using the following equation [20]: the formula magnitude vector (MV)90 = m (sin2α - cos2α) was used to represent the vector size along the axis of 90°, where m denotes astigmatism (diopters) and α is the astigmatism meridian (degrees), indicating the horizontal and vertical components in refraction. To calculate the difference in diopter components along the 135° and 45° axes, the equation MV135 = m(sin2(α-45) - cos2(α-45)) was applied. To preserve the SE format, the MV components were halved.

The data were analyzed using R software [21]. Univariate statistics were performed by calculating the change in SE values, which were obtained by subtracting the subjective clinical refraction (SCR) SE value from the QuickSee SE value. An equivalent method was performed to MV90/MV135. A positive change indicated that QuickSee produced higher estimates for the respective values. As the data did not follow a normal distribution, as determined by the Shapiro-Wilk test, comparisons were made using the Wilcoxon signed-rank test to assess whether the differences significantly deviated from zero. Bivariate and trivariate analyses were conducted using Hotelling’s test. A p-value of less than 0.05 was considered statistically significant.

## Results

The study population included 368 eyes from 368 healthy Brazilian public preschool and elementary school children, with 180 (49%) being male and 188 (51%) female. The average age was 5.7 ± 0.91 years. Among the children, 113 (30.7%) had a monocular VA without correction with the worst vision ≤0.7, while 255 (69.3%) had a monocular VA without correction with the worst vision >0.7. A total of 98 eyeglasses were prescribed and provided to 98 (26.63%) children.

Table 2 shows the discrepancies in the values of refraction measured by QuickSee and subjective clinical refraction under cycloplegia. The mean ± standard deviation (SD) values for QuickSee were SE +0.38±0.60 D, MV90 +0.20 ± 0.33 D, and MV135 +0.03 ± 0.22 D. The results suggest that QuickSee slightly overestimated both spherical and cylindrical powers, though this variation was not clinically relevant. The most significant difference was observed in the SE, which was under half a diopter.

**Table 2:** Univariate analysis of differences between RE refraction values obtained under cycloplegia by QS and SCR in 368 healthy Brazilian preschool and elementary school children with limited access to ocular health care. SE: spherical equivalent; SD: standard deviation; MV90: magnitude vector on the 90° axis; MV135: discrepancy between the diopter components projected onto the 135° axis and the 45° axis; QS-SCR: discrepancy in values of refraction obtained by QuickSee handled autorefractor and subjective clinical refraction, both under cycloplegia; RE: right eye; *: Wilcoxon signed-rank test; **: statistically significant results with a p-value less than 0.05

	Variables	Average	DP	P-value*
QS-SCR	SE	+0.38	0.60	<0.0001**
	MV90	+0.20	0.33	<0.0001**
	MV135	+0.03	0.22	0.0395**

**Figure 1:**
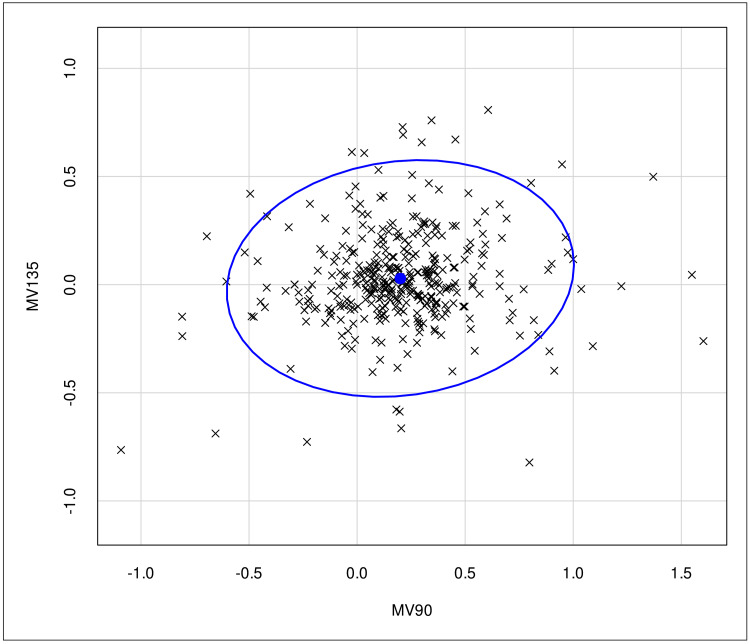
A bivariate statistical analysis of the influence of the parameters MV90 and MV135 on the discrepancies in RE refraction values obtained under cycloplegia by QS and SCR in 368 healthy Brazilian preschool and elementary school children with limited access to eye care. MV90: magnitude vector on the 90° axis; MV135: discrepancy between the diopter components projected onto the 135° axis and the 45° axis; RE: right eye; QS: QuickSee; SCR: subjective clinical refraction

Figure 1 illustrates the bivariate analysis conducted to evaluate the impact of astigmatism on the discrepancies in refraction values between QuickSee and subjective clinical refraction under cycloplegia.

**Figure 2:**
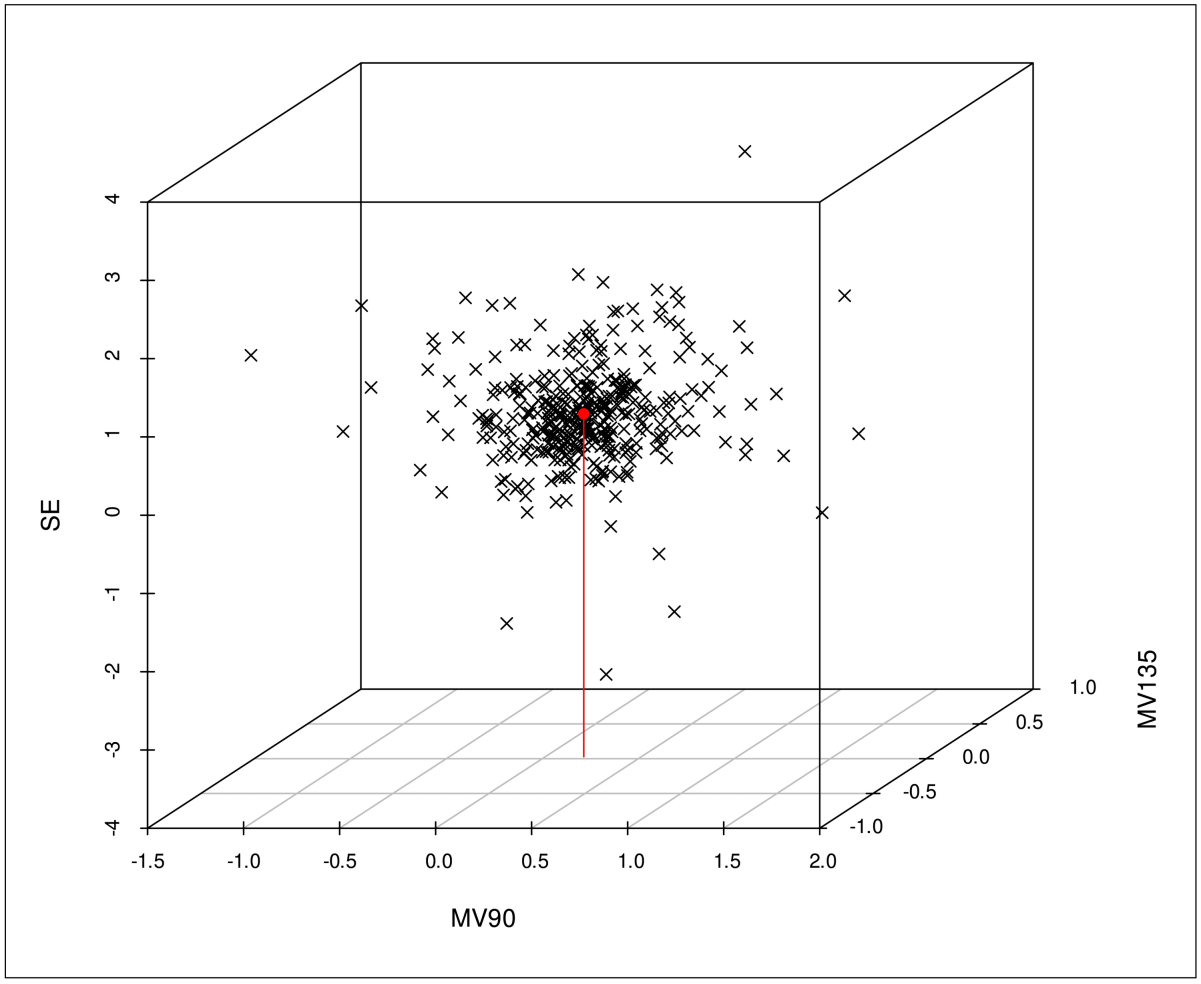
A trivariate statistical analysis demonstrating the influence of the parameters SE, MV90, and MV135 on the discrepancies in RE refraction values measured under cycloplegia by QS and SCR. MV90: magnitude vector on the 90° axis; MV135: discrepancy between the diopter components projected onto the 135° axis and the 45° axis; RE: right eye; QS: QuickSee; SCR: subjective clinical refraction

Figure 2 presents a trivariate analysis using a three-dimensional plot to explore the relationship between SE, MV90, and MV135, and their impact on the discrepancies in RE refraction measured by QuickSee and subjective clinical refraction under cycloplegia.

Converting refraction values from vector sizes to the conventional format revealed an average discrepancy of +0.58 to -0.40 × 40 (SE of +0.38 D) between QuickSee and subjective clinical refraction for the RE of each participant.

## Discussion

In the study, 113 (30.70%) of the 368 children in preschool and elementary school had monocular VA without correction worse ≤0.7. A total of 98 glasses were prescribed, i.e., 26.6% of the school children examined received an optical prescription. One of the possible explanations lies in the lack of programs for large-scale visual care of school children in areas of care gaps, which contributed to the inclusion in the study of school children identified with visual difficulties and/or low school performance.

The comparison of the differences between the values obtained from the refraction of the right eye by QuickSee, under cycloplegia, and the values of the subjective clinical refraction, under cycloplegia, showed that the mean difference in the magnitude of the refractive error in SE was 0.38 ± 0.60D (p < 0.0001). The mean differences in meridians 90° and 135° were 0.20 ± 0.33D (p < 0.0001) and 0.03 ± 0.22D (p = 0.0395), respectively (Table 2). The mean difference of refraction in SE within 0.50 D is considered of no clinical relevance [13-16]. The advantages of QuickSee, a tabletop autorefractor, include being a compact, light, easy-to-use autorefractor with great portability, favoring that the student identified in visual screening can be submitted to refractometry at the school itself, instead of being referred to another service, thus reducing absenteeism. Community projects focused on the eye health of school children have often encountered absenteeism, which can lead to unnecessary expenses and missed opportunities for children to receive important examinations [22]. Socioeconomic and cultural conditions made it difficult for students identified in visual screening at school to access ophthalmological examinations [23,24].

One study evaluated the performance of QuickSee in diagnosing refractive errors in 123 school-age children (9.9 ± 3.3 years) with moderate refractive errors [15]. The children underwent autorefraction with a tabletop autorefractor (Topcon KR 8800, Topcon Corporation, Tokyo, Japan), QuickSee, and subjective clinical refraction. QuickSee measurements were performed in 62 children without cycloplegia and 61 children with cycloplegia. The refraction of QuickSee by mean SE agreed within 0.50 D of the subjective clinical refraction in 71% (without cycloplegia) and 70% (under cycloplegia) of the cases. The agreement between the tabletop autorefractor and the subjective clinical refraction for the same threshold was 61% (without cycloplegia) and 77% (under cycloplegia). The elevated level of agreement between QuickSee and subjective clinical refraction, along with the VA improvement achieved in both study groups using QuickSee, suggest that QuickSee is a valuable tool for assessing refraction in school-age children [15].

Another study evaluated the performance of the QuickSee with a +2.00 D fogging lens to determine whether viewing through the fogging lenses resulted in more accurate detection of hyperopia [16]. The QuickSee measurements with and without the fogging lenses were compared among 53 children with any level of hyperopia. Refractive error values obtained with +2.00 D lenses were adjusted by subtracting 2 D before comparison. The difference between the QuickSee measurements without versus with the +2.00 D fogging lenses was -0.34 to 0.17 (p = 0.51) [16].

Bivariate analysis indicated that astigmatism had minimal impact on the results, with a more pronounced effect observed for the MV90 vector than the MV135 vector. These findings are consistent with the study reported by Naeser, which showed more significant differences in the vertical and horizontal components compared to the oblique component. Naeser concluded that the vertical component was more affected by eyelid tension and the blinking mechanism [20].

The trivariate analysis highlighted the influence of refractive error magnitude on both the SE and the meridians at 90° and 135°. It also showed that converting refraction into vector values in the conventional format resulted in an average difference of +0.58 D between QuickSee and subjective clinical refraction, with a deviation of -0.40 DC of the 40° axis to the RE of each participant.

In this study, QuickSee proved to be a straightforward and efficient method for refractometry. Clinically, QuickSee produced measurements similar to those from subjective clinical refraction, supporting its use as a complementary tool for estimating refraction, particularly in settings where standard clinical exams are challenging to conduct.

Limitations

This study on the QuickSee autorefractor in Brazilian children presents several limitations that affect its generalizability. The sample was restricted to a narrow age group and socioeconomic context, and the low prevalence of severe refractive errors limited the evaluation of the device across its full range. The exclusive use of cycloplegia-induced measurements and analysis of only the RE may not fully reflect real-world conditions or bilateral variability. Additionally, QuickSee’s slight overestimation of refractive values and the lack of long-term follow-up data leave questions about its broader effectiveness and impact on visual health outcomes.

## Conclusions

The low prevalence of significant refractive errors in the study population restricted a comprehensive evaluation of QuickSee’s performance across its full measurement range. However, a notable strength of the study was the on-site evaluation of children at their schools, which mitigated the high absenteeism rates typically associated with referrals for ophthalmic assessments. This approach enhances the study’s relevance in Brazilian public ocular health.
